# Mathematical Modeling for the Pathogenesis of Alzheimer's Disease

**DOI:** 10.1371/journal.pone.0015176

**Published:** 2010-12-14

**Authors:** Ishwar K. Puri, Liwu Li

**Affiliations:** 1 Department of Engineering Science and Mechanics, Virginia Polytechnic Institute and State University, Blacksburg, Virginia, United States of America; 2 Department of Biological Sciences, Virginia Polytechnic Institute and State University, Blacksburg, Virginia, United States of America; Dana-Farber Cancer Institute, United States of America

## Abstract

Despite extensive research, the pathogenesis of neurodegenerative Alzheimer's disease (AD) still eludes our comprehension. This is largely due to complex and dynamic cross-talks that occur among multiple cell types throughout the aging process. We present a mathematical model that helps define critical components of AD pathogenesis based on differential rate equations that represent the known cross-talks involving microglia, astroglia, neurons, and amyloid-β (Aβ). We demonstrate that the inflammatory activation of microglia serves as a key node for progressive neurodegeneration. Our analysis reveals that targeting microglia may hold potential promise in the prevention and treatment of AD.

## Introduction

Alzheimer's disease (AD) is one of the most prevalent neurodegenerative disorders associated with aging, causing dementia and related severe public health concerns [1]. Despite extensive research effort and progress, the pathogenesis of AD remains incompletely understood, partly due to highly complex and intertwined intercellular cross-talks taking place throughout the aging process [2]. Consequently, despite limited treatment options to manage and slow the progression of AD, no effective cure is available.

**Figure 1:**
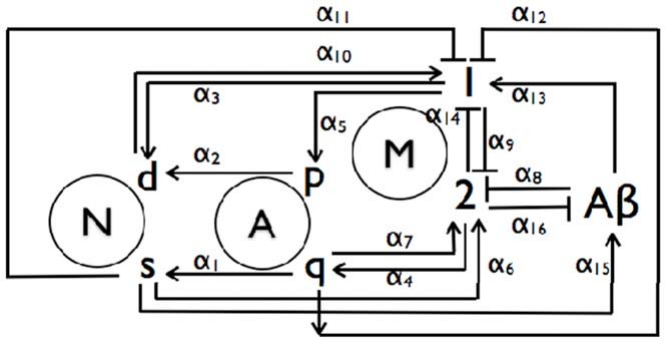
Schematic of the AD mechanism that incorporates feedback influences from surviving and dead neurons, N_s_ and N_d_, quiescent and proliferating astroglia A_q_ and A_p_, reactive and normal microglia, M_1_ and M_2_, and Aβ. The rates associated with the pathways are included in Table 1.

Although the deposition of amyloid-β (Aβ) peptides and formation of senile plaques in the brain is the cardinal morphological feature identifying the clinical phenotype of AD [3], [4], increasing clinical and basic studies suggest that inflammatory activation of microglia may play an equally important role during the initiation and progression of the disease [5]. Microglia are resident innate immune macrophages within brain tissues, capable of expressing pro-inflammatory mediators and reactive oxygen species when activated by inflammatory signals including amyloid-β (Aβ) [6]. In healthy brains, together with quiescent astroglia (Aq), resting microglia may adopt an anti-inflammatory state (M2) and in turn foster neuron survival (Ns) and prevent astroglia proliferation (Ap) [7], [8]. As inflammatory signals (e.g. Aβ) gradually build, microglia may adopt an activated pro-inflammatory state (M1), leading to A_p_ proliferation and neuron death (Nd) [9], [10], [11]. Neuronal debris, amyloid-β (Aβ), and/or proliferating astroglia (Ap) may in turn further exacerbate the inflammatory phenotype of M1 macroglia [12], [13]. The multiple positive and negative feedbacks among these cells are thus crucial for neurodegeneration that eventually alters the neuronal structure and function during the pathogenesis of AD (Figure 1).

**Table 1:** Mathematical parameters describing the functional interactions among various cell types.

Rate	1/year	Pathway	S(N_s_)	S(N_d_)	S(M_1_)	S(M_2_)	Sensitivity
α_1_	10^−5^	A_q_ → N_s_	50000	−50000	−6000	6000	Strong
α_2_	10^−3^	A_p_ → N_d_	−500	500	−60	60	Weak
α_3_	10^−2^	M_1_ → N_d_	−200	200	−35	35	Weak
α_4_	10^−4^	M_2_ → A_q_	500	−500	150	−150	Weak
α_5_	10^−2^	M_1_ → A_p_	−3	3	1	−1	Weak
α_6_	10^−2^	N_s_ → M_2_	500	−500	−6000	6000	Weak
α_7_	10^−4^	A_q_ → M_2_	5000	−5000	−50000	50000	Strong
α_8_	10^−2^	Aβ ⊥ M_2_	−400	400	5000	−5000	Moderate
α_9_	10^−2^	M_1_ ⊥ M_2_	−30	30	250	−250	Weak
α_10_	10^−2^	N_d_ → M_1_	−8	8	90	−90	Weak
α_11_	10^−2^	N_s_ ⊥ M_1_	500	−500	−6000	6000	Moderate
α_12_	10^−4^	A_q_ ⊥ M_1_	5000	−5000	−50000	50000	Strong
α_13_	10^−2^	Aβ → M_1_	−400	400	5000	−5000	Moderate
α_14_	10^−4^	M_2_ ⊥ M_1_	5000	−5000	−50000	50000	Strong
α_15_	1	N_s_ → Aβ	−10	10	100	−100	Weak
α_16_	10^−2^	M_2_ ⊥ Aβ	100	−100	−1000	1000	Weak
α_r_	1	M_2_ ⊥ Aβ	8	−8	−100	100	Weak

The rates α_i_ associated with the pathways of the AD mechanism, and the sensitivities of the N_s_ and N_d_ populations to variations in the values of α_i_. The values for the sensitivity coefficients S(N_j, j = s, d_)  =  dN_j_/dα_i_ are determined after 20 years for ±2.5% perturbations in each α_i_ value. A cell population is more sensitive to a change in a rate that produces a larger value of |S(N_j_)|. Positive S(N_j_) imply that a rate contributes to an increase in N_j_ while a negative value entails a corresponding population decrease.

Due to its multi-cellular components and complex nature, conventional experimental approaches have failed to identify critical underlying causes for AD, contributing to the lack of an effective therapeutic treatment. Mathematical models can serve as powerful tools to understand the molecular and cellular processes that control complex diseases [14], [15]. Indeed, there have been several attempts to model the process of senile plaque formation [16], [17], [18], [19]. Specifically, these approaches focused on a nucleation step that is coupled with rates for the irreversible binding of Aβ monomers to the fibril ends, the lateral aggregation of filaments into fibrils, and fibril elongation through end-to-end association. Other modeling efforts examined the signaling cascade responsible for microglia migration and activation in response to an initial inflammation-provoking stimulus involving Aβ [16], [20].

However, no systematic modeling approaches have been reported to examine the network cross-talks among microglia, neuron, and astroglia, and the corresponding pathological consequence. Here, we evaluate the dynamic network involving multiple cross-talks among distinct states of microglia, astroglia, and neurons through a mathematical model. Our approach has led to an intriguing insight suggesting that microglia activation in addition to a threshold for Aβ may be the critical initiator for the pathogenesis of AD.

## Methods

### Mathematical Method

We propose a sixteen pathway AD mechanism involving seven species that is shown schematically in Fig. 1. The paths have rates α_i_ that implicitly represent the influences of intercellular signaling along them. The mechanism is based on an assumption of constant risk of neuronal death, i.e., a single event randomly initiates cell death independently of the state of any other neuron at any instant [21]. The spatiotemporal influence of diffusion is neglected since local cell events are assumed to occur on a slower timescale than signal dispersion through chemotaxis.

The seven rate equations for the cell populations and the number of Aβ molecules in an arbitrary local volume can be written through seven coupled rate equations, namely,
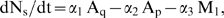
(1)

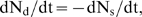
(2)

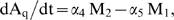
(3)

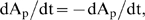
(4)

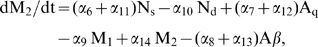
(5)

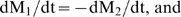
(6)

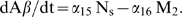
(7)


These relate the change in each cell population or the number of Aβ molecules at any instant to the values of all species at that time. For instance, Eq. (1) relates the rate of change in N_s_ to the A_q_, A_p_, and M_1_ populations with the pathway weights α_1_, α_2_, and α_3_, respectively. Whereas A_q_ increases the rate of change of N_s_, A_p_ and M_1_ decrease it. Equation (5) for the rate of change of the M_2_ population is the most complex, since it involves nine pathway weights, and five cell populations and Aβ. The conversion of N_s_ into N_d_ is irreversible, whereas those of A_q_ and M_2_ into A_p_ and M_1_ are reversible.

The rates for each α_i_ are specified, as shown in presented in Table 1 for each pathway. Since the literature points to the path N_s_ → Aβ being dominant, we assume that it is also the fastest. Its rate is set at 1/year, i.e., each year every N_s_ cell stimulates the formation of a sustaining an Aβ molecule. Likewise, since neuronal survival decreases significantly once disease progresses, we assume that the overall path M_2_ → A_q_ → N_s_ is slow so that the associated rates α_1_ and α_4_ are also relatively the smallest. The other rates are similarly specified in terms of their relative abilities to facilitate or inhibit the formation of a cell or Aβ molecule according to the particular pathway. Next, we specify the initial composition of the volume under consideration. These initial conditions for the seven species are presented in Table 2.

**Table 2:** Sensitivities of the cell types to the initial conditions.

	N_s_(0)	N_d_(0)	A_q_(0)	A_p_(0)	M_1_(0)	M_2_(0)	Aβ (0)
**Value**	10^4^	10^2^	10^5^	10^3^	10^3^	10^5^	10^3^
**S(N_s_)**	1	≈0	≈0	≈0	−0.2	0	≈0
**S(N_d_)**	≈0	1	≈0	≈0	0.2	≈0	≈0
**S(A_q_)**	≈0	≈0	1	≈0	−0.2	≈0	≈0
**S(A_p_)**	≈0	≈0	≈0	1	0.2	≈0	≈0
**S(M_1_)**	≈0	0.2	≈0	≈0	1.2	≈0	≈0
**S(M_2_)**	≈0	−0.2	≈0	≈0	−0.2	1	≈0
**S(Aβ)**	1	≈0	≈0	≈0	−0.2	≈0	≈0

Initial values X(0) of the initial cell populations and the number of molecules of Aβ in an arbitrary local volume and orders of magnitude for the sensitivity coefficients S(N_j, j = s,d_)  =  dN_j_/d(X(0)) determined after 20 years for tenfold perturbations, i.e., 10× and 10^−1^×, in these initial values.

## Results

Our objective is to be able to describe neuropathogenesis during AD in terms of the N_s_ and N_d_ populations. Hence, we first determine the sensitivities of these cells to changes in the rates α_i_, using the usual definition of the sensitivity coefficient,
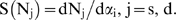
(8)


The sensitivity coefficients for N_s_, N_d_, M_1_ and M_2_ cells, presented in Table 1, are determined after 20 years for ±2.5% perturbations in each α_i_ value. A cell population is more sensitive to a change in a rate that produces a larger value of |S(N_j_)|. Positive values for S(N_j_) imply that a rate contributes to an increase in N_j_ while a negative value implies that its influence leads to a corresponding population decrease. The sensitivity analysis shows that the N_s_ and N_d_ populations are most sensitive to the path A_q_ → N_s_, which increases neuronal survival and decreases neuron death. Important paths that inhibit neuropathogenesis include A_q_ → M_2_, A_q_ ⊥ M_1_ and M_2_ ⊥ M_1_, while those that enhance disease involve M_1_ → N_d_, Aβ ⊥ M_2_ and Aβ → M_1_.

A similar analysis that perturbs the initial cell populations and the number of Aβ molecules tenfold is presented in Table 2. It shows that, in comparison to the other species, the M_1_ population is most sensitive to these substantial perturbations in the initial amount of any species while N_d_ is only sensitive to the initial amounts of M_1_ and M_2_. This implies an important role for microglia during AD progression. The sensitivity coefficients S(M_1_) and S(M_2_), also presented in Table 1, show that, as for N_s_ and N_d_, the dominant paths that inhibit neuropathogenesis by affirming M_2_ and decreasing M_1_ are also A_q_ → M_2_, A_q_ ⊥ M_1_ and M_2_ ⊥ M_1_. Once again, paths 7 and 14 involving Aβ, i.e., Aβ ⊥ M_2_ and Aβ → M_1_ promote AD progression. The model suggests that interventions aimed at decreasing α_8_ and α_13_, which involve M_1_, M_2_ and Aβ and contribute to AD progression, are the ones more likely to diminish neuropathogenesis. This intuitive result emphasizes that decreasing the number of reactive microglia and ensuring a sufficient population of quiescent astroglia is important in treating AD.

**Figure 2:**
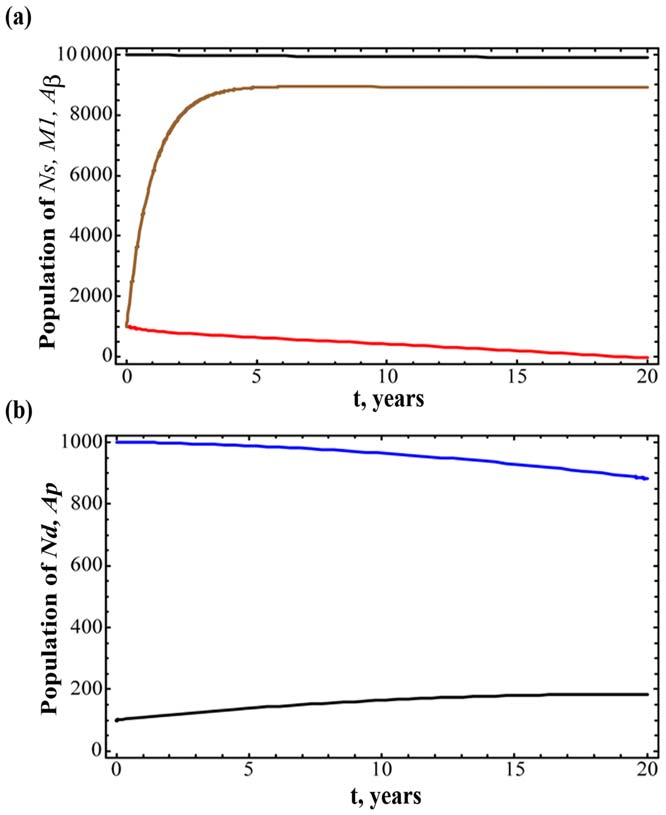
Dynamic simulation of various cell populations during the progression of Alzheimer's disease. The (a) N_s_ (black), M_1_ (red) and Aβ (blue), and (b) N_d_ (black) and A_p_ (blue) populations over 20 years for the rates reported in Table 1. The removal rate α_r_ stabilizes the net number of Aβ molecules after three years so that there is only a gradual increase in N_d_ and corresponding decline in N_s_ thereafter. The microglia populations are also consequently relatively stable.

The temporal variation in various species for the rates in Table 1 is illustrated in Fig. 2. Figure 2(a) presents the N_s_, M_1_ and Aβ populations over 20 years, and Fig. 2(b) the corresponding values for N_d_ and A_p_. Most notable is the influence of the removal rate α_r_, which stabilizes the number of Aβ molecules after three years. Following that period, there is only a gradual increase in N_d_ that is coupled with a corresponding decline in N_s_. Consequently, the microglia populations are also relatively stable. Therefore, the rates in Table 1 should be considered as being representative of a healthy population.

**Figure 3:**
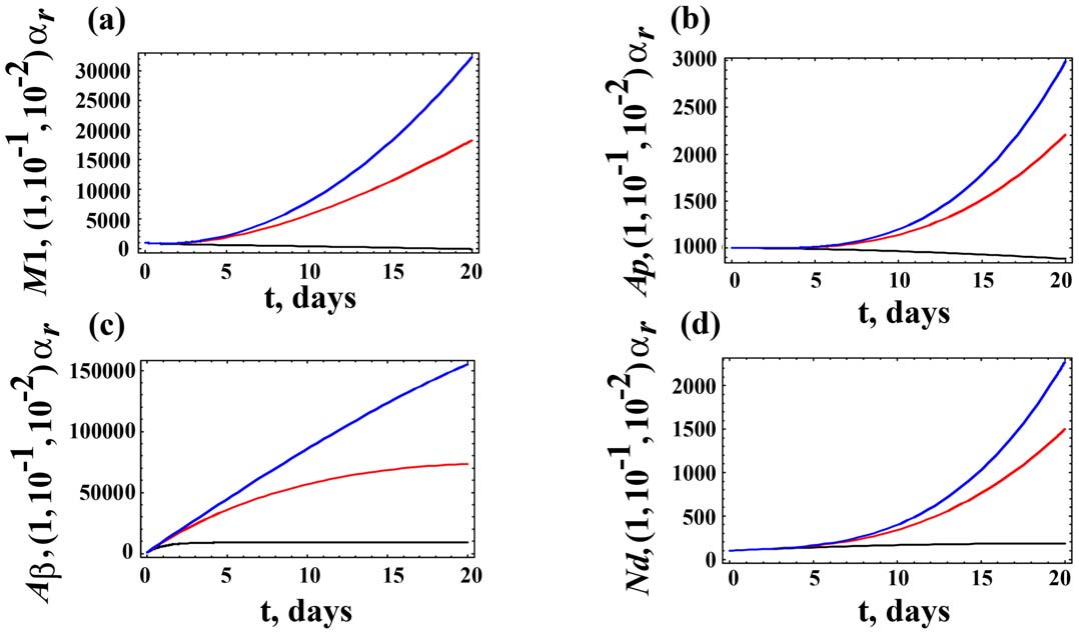
Dynamic variations of cell populations given distinct Aβ removal rate. Variations in the (a) M_1_, (b) A_p_, (c) Aβ, and (d) N_d_ populations over 20 years for three values of α_r_  =  1× (black), 10^−1^× (red), and 10^−2^× (blue) the value reported in Table 1 for the Aβ removal rate. As α_r_ decreases, there is an increase in neuropathogenesis so that all four populations increase.

We examine the influence of varying α_r_ on neuropathogeneis in Fig. 3, which presents the M_1_, A_p_, Aβ and N_d_ populations over 20 years for three values of α_r_. As α_r_ decreases, there is an increasing neuronal death. Thus, all four populations, which are associated with AD progression, increase. While microglia play an important role in AD, Fig. 3 shows how the local Aβ concentration plays a critical role in initiating and promoting AD.

**Figure 4:**
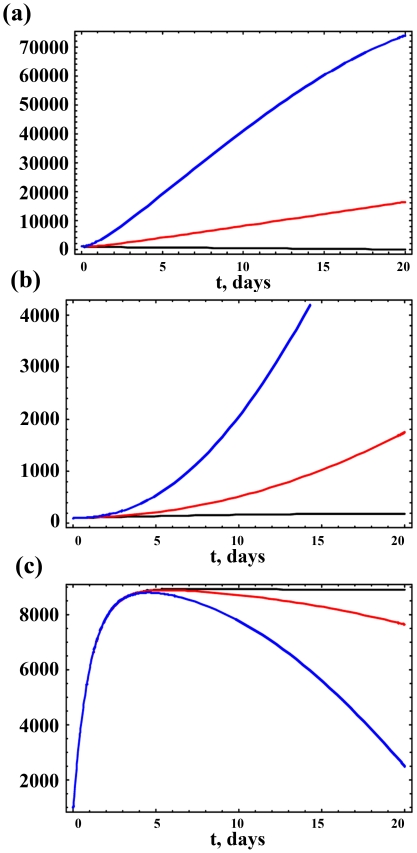
Dynamic variations of cell populations given distinct impacts of Aβ on M1 macrophages (the value of α_13_). Variations in the (a) M_1_, (b) N_d_ and (c) Aβ populations over 20 years for three values of α_13_  =  1× (black), 10× (red), and 50× (blue) the value reported in Table 1 for the path Aβ → M_1_. As α_13_ increases, M_1_ and N_d_ also increase and, consequently, there is an associated decrease in neuronal survival. This is also illustrated through Eqs. (1) and (2) of the mathematical model.

We investigate this further by varying α_8_ and α_13_. Figure 4 presents results for the M_1_, N_d_ and Aβ populations over 20 years for three values of α_13_. As α_13_ increases, the M_1_, A_p_ and N_d_ populations also increase, leading to an associated decrease in neuronal survival, as illustrated through Eqs. (1) and (2) of the mathematical model. A tenfold increase in α_13_ leads to a near doubling in N_d_ after 20 years. As N_s_ decreases so does Aβ, but the smaller protein concentration is still sufficient to promote neuropathogenesis among the smaller N_s_ population. Identical results are obtained for similar variations in the rate α_8_ for Aβ ⊥ M_2_, since the sensitivity coefficients for each of M_1_ and N_d_ towards paths 8 and 13 are identical.

## Discussion

We present a mathematical model for neuropathogenesis during AD that involves neurons, normal and reactive glial cells, and Aβ. It uses neuronal death as a surrogate for senile plaque formation. By monitoring neuronal health, we are able to identify intuitive strategies for interventions. In particular, the model suggests that the most effective intervention is one that improves the inhibition of reactive microglia and Aβ by normal microglia, and ensuring a sufficient population of quiescent astroglia. Overall, neuropathogenesis proceeds through the production of reactive microglia.

Our analysis is consistent with experimental data that indicate that inflammation may be an early initiator for AD, long before the apparent senile plaque formation [22], [23]. It further reinforces the notion that additional studies should be directed at examining earlier inflammatory signals and alterations involving microglia as a key node so as to better define AD initiation and understand mechanisms for effective prevention and treatment of the disease.

We realize that our mathematical analysis is an initial attempt to examine AD and may not fully account for the associate intertwined cellular communication pathways. Nevertheless, it serves as a hypothesis provoking and building process that should encourage integrated analyses of AD pathogenesis. Future experimental data examining the cross-talks among microglia, astroglia, and neurons will allow us to better refine our model and implement realistic parameters in the rate equations.

## References

[1] Fotuhi M, Hachinski V, Whitehouse PJ (2009). Changing perspectives regarding late-life dementia.. Nat Rev Neurol.

[2] Citron M (2010). Alzheimer's disease: strategies for disease modification.. Nat Rev Drug Discov.

[3] Selkoe DJ (1997). Neuroscience - Alzheimer's disease: Genotypes, phenotype, and treatments.. Science.

[4] Holtzman DM, Bales KR, Tenkova T, Fagan AM, Parsadanian M (2000). Apolipoprotein E isoform-dependent amyloid deposition and neuritic degeneration in a mouse model of Alzheimer's disease.. Proceedings of the National Academy of Sciences of the United States of America.

[5] Perry VH, Nicoll JA, Holmes C (2010). Microglia in neurodegenerative disease.. Nat Rev Neurol.

[6] Lue LF, Kuo YM, Beach T, Walker DG (2010). Microglia activation and anti-inflammatory regulation in Alzheimer's disease.. Mol Neurobiol.

[7] Kigerl KA, Gensel JC, Ankeny DP, Alexander JK, Donnelly DJ (2009). Identification of two distinct macrophage subsets with divergent effects causing either neurotoxicity or regeneration in the injured mouse spinal cord.. J Neurosci.

[8] Neumann J, Sauerzweig S, Ronicke R, Gunzer F, Dinkel K (2008). Microglia cells protect neurons by direct engulfment of invading neutrophil granulocytes: a new mechanism of CNS immune privilege.. J Neurosci.

[9] Pang Y, Campbell L, Zheng B, Fan L, Cai Z (2010). Lipopolysaccharide-activated microglia induce death of oligodendrocyte progenitor cells and impede their development.. Neuroscience.

[10] Park KW, Baik HH, Jin BK (2008). Interleukin-4-induced oxidative stress via microglial NADPH oxidase contributes to the death of hippocampal neurons in vivo.. Curr Aging Sci.

[11] Brown GC, Neher JJ (2010). Inflammatory neurodegeneration and mechanisms of microglial killing of neurons.. Mol Neurobiol.

[12] Mandrekar-Colucci S, Landreth GE (2010). Microglia and inflammation in Alzheimer's disease.. CNS Neurol Disord Drug Targets.

[13] Cameron B, Landreth GE (2010). Inflammation, microglia, and Alzheimer's disease.. Neurobiol Dis.

[14] Edelstein-Keshet L (2005). Mathematical Models in Biology: Society for Industrial and Applied Mathematics.

[15] Ganguly R, Puri IK (2006). Mathematical model for the cancer stem cell hypothesis.. Cell proliferation.

[16] Edelstein-Keshet L, Spiros A (2002). Exploring the Formation of Alzheimer's Disease Senile Plaques in Silico.. Journal of Theoretical Biology.

[17] Lomakin A, Teplow DB, Kirschner DA, Benedek GB (1997). Kinetic Theory of Fibrillogenesis of Amyloid Œ≤ -protein.. Proceedings of the National Academy of Sciences of the United States of America.

[18] Pallitto MM, Murphy RM (2001). A Mathematical Model of the Kinetics of [beta]-Amyloid Fibril Growth from the Denatured State.. Biophysical Journal.

[19] Lee C-C, Nayak A, Sethuraman A, Belfort G, McRae GJ (2007). A Three-Stage Kinetic Model of Amyloid Fibrillation.. Biophysical Journal.

[20] Luca M, Chavez-Ross A, Edelstein-Keshet L, Mogilner A (2003). Chemotactic signaling, microglia, and Alzheimer's disease senile plaques: Is there a connection?. Bulletin of Mathematical Biology.

[21] Clarke G, Collins RA, Leavitt BR, Andrews DF, Hayden MR (2000). A one-hit model of cell death in inherited neuronal degenerations.. Nature.

[22] Dudal S, Krzywkowski P, Paquette J, Morissette C, Lacombe D (2004). Inflammation occurs early during the Abeta deposition process in TgCRND8 mice.. Neurobiol Aging.

[23] Cuello AC, Ferretti MT, Leon WC, Iulita MF, Melis T (2010). Early-stage inflammation and experimental therapy in transgenic models of the Alzheimer-like amyloid pathology.. Neurodegener Dis.

